# Phytoremediation Potential of the Invasive Plant *Datura stramonium* (Solanaceae) for Toxic Metal Removal from Soil in the Qinghai–Tibet Plateau

**DOI:** 10.3390/biology15100807

**Published:** 2026-05-19

**Authors:** Ngawang Bonjor, Taican Huang, Xinyi Luan, Zhou Hui, Xin Tan, La Qiong, Junwei Wang

**Affiliations:** 1 Key Laboratory of Biodiversity and Environment on the Qinghai-Tibetan Plateau, Ministry of Education, School of Ecology and Environment, Xizang (Tibet) University, Lhasa 850000, China; bonjor20010610@163.com (N.B.);; 2 Yani Wetland Ecosystem Positioning Observation and Research Station, Nyingchi 860000, China

**Keywords:** Qinghai–Tibet Plateau, mining-contaminated soil, toxic metals, *Datura stramonium*, phytoremediation

## Abstract

The invasive plant *Datura stramonium* L. possesses strong reproductive capacity and ecological adaptability, showing a tendency to spread rapidly, especially in highly human-disturbed habitats. To explore its resource utilization pathway—turning waste into wealth—and to address toxic metal pollution in strongly human-disturbed areas (such as mining regions), this study evaluates its phytoremediation potential in contaminated soils on the Qinghai–Tibet Plateau. We established a non-planted control and three planting density treatments to compare the removal rates of Pb, Cd, Cr, and As. To our knowledge, this is the first study to assess how planting density influences the multi-metal phytoremediation performance of this invasive species in a high-altitude plateau environment. The results showed that planting significantly increased toxic metal removal rates, with overall efficiency generally improving at higher densities, particularly for Cr. Analysis of bioconcentration and translocation factors revealed distinct element-specific accumulation patterns. Pb and As were primarily enriched and retained in the roots. Interestingly, while Cd exhibited a strong localized tendency to accumulate in the leaves, its overall root-to-shoot translocation remained relatively restricted at the whole-plant level, similar to Cr. Overall, *D. stramonium* functions primarily through root stabilization for Pb, As, and Cr, alongside partial aboveground accumulation for Cd. However, given its toxic and invasive nature, any practical phytoremediation application requires strict post-harvest biomass management and ecological monitoring to prevent secondary spread.

## Simple Summary

Mining activities on the high-altitude Qinghai–Tibet Plateau have led to soil contamination with harmful toxic metals. This study investigated whether the highly adaptable, yet invasive, plant *Datura stramonium* can be used to clean up these polluted soils. We discovered that planting the plants closer together (higher density) significantly boosts their overall ability to remove metals from the soil, particularly chromium. The plant handles different metals in unique ways: it traps lead and arsenic in its roots, pushes cadmium up into its leaves, and largely restricts chromium to its roots. While *Datura stramonium* L. shows strong potential as a tool for restoring toxic metal-polluted mining sites, its toxic and invasive nature means that any real-world cleanup efforts must include strict harvesting and disposal management to prevent further ecological harm.

## 1. Introduction

The Qinghai–Tibet Plateau is characterized by high elevation, harsh climatic conditions, a short growing season, weakly developed soils, and slow ecosystem recovery, and is therefore widely recognized as a fragile region highly sensitive to anthropogenic disturbance [1,2]. In recent years, intensified mineral exploitation, infrastructure construction, and urban expansion have increased local inputs and accumulation risks of toxic metals in soils. In particular, in areas affected by mining activities and tailing storage, potentially toxic elements such as Pb, Cd, Cr, and As can be transported into surrounding soils via dust deposition, surface runoff, and leachate migration, thereby altering vegetation performance, soil microbial communities, and the safety of agro-pastoral products [2,3,4,5]. Previous studies across the Qinghai–Tibet Plateau have documented heavy-metal enrichment and associated ecological risks in soils, identifying anthropogenic sources—especially industrial and mining-related inputs—as major contributors. Investigations of the soil–water–food system have further highlighted that As, Cd, Cr, and Pb may pose human health risks through food-chain transfer [2,6]. Therefore, developing efficient, low-disturbance, and sustainable remediation technologies for contaminated soils—particularly in cold, high-altitude mining regions—is of significant ecological and societal importance.

Among the various remediation approaches, phytoremediation has attracted substantial attention because it is comparatively cost-effective, environmentally friendly, and can be implemented in parallel with vegetation restoration [7,8]. Seminal studies proposed that plants can remediate contaminated sites by absorbing and accumulating pollutants in harvestable tissues or by reducing metal mobility and bioavailability through rhizosphere processes, often in concert with microbially mediated transformations that improve soil quality [9,10]. However, the application of phytoremediation in mining areas remains challenging. High metal loads, nutrient-poor substrates, degraded soil structure, abnormal pH and salinity, and intense wind and water erosion can all constrain plant establishment and biomass production, thereby limiting remediation efficiency [7,11,12]. These constraints are particularly pronounced in high-altitude environments, where successful restoration relies on stress-tolerant plant materials capable of surviving, establishing persistent vegetative cover, and on appropriate agronomic management strategies [7].

Invasive plants are currently exhibiting an expansion trend in plateau ecosystems. Plateau-scale assessments indicate that, under the combined influences of climate warming and the corridor effects associated with intensified human activities, multiple alien invasive species face an increased risk of expanding their potential suitable habitats across the Qinghai–Tibet Plateau, and this risk may further intensify in the future [13]. Invasive plants often possess strong resource-acquisition capacity and high tolerance to environmental stress, enabling rapid establishment and dominance in mining sites and other disturbed habitats. Such traits may provide “highly adaptable plant materials” for remediation, but they also entail ecological risks and can raise management controversies. Consequently, the use of invasive plants for remediation of contaminated soils requires cautious evaluation and strict control to balance remediation benefits against the risk of further spread.

*Datura stramonium* L., native to the Americas, is widely recognized as an invasive plant that has expanded into plateau areas such as Tibet [14,15]. This species possesses strong reproductive capacity and ecological adaptability, showing a high tendency to spread, especially in highly human-disturbed habitats. To explore its resource utilization pathway—turning waste into wealth—and to address toxic metal pollution in strongly human-disturbed areas (such as mining regions), this study attempts to utilize this invasive plant for the phytoremediation of contaminated soils. Recent studies have analyzed the invasion mechanisms and potential distribution risks of *D. stramonium* in Tibet from climatic and niche perspectives, emphasizing the need to enhance early warning and dispersal control [14]. Concurrently, plants of the genus *Datura* exhibit strong stress tolerance and characteristics of secondary metabolite accumulation, such as tropane alkaloids. They can grow in various adverse soil conditions and are considered to possess potential for tolerating and accumulating multiple metals (metalloids) [16]. These attributes lend the species candidate value in scenarios involving the remediation of contaminated soils in high-altitude mining areas. During the early stages of ecological restoration, if *D. stramonium* can rapidly establish ground cover and achieve absorption/immobilization of target metals, it may simultaneously serve functions such as dust suppression, soil stabilization, risk mitigation, and promotion of soil improvement [11].

A foundation has been established regarding the remediation potential of *D. stramonium* in toxic metal-contaminated soils. For example, studies have shown that the application of chitosan/chitosan nanoparticles can enhance the remediation of Cd-contaminated soil by *D. stramonium* and improve its metal translocation efficiency, indicating a modifiable remediation potential [17]. For Cr (VI)-contaminated soil, research has also investigated the enhancing mechanisms of chelation/conditioning agents such as EDTA and organic acids on the remediation process by *D. stramonium*, providing a basis for optimization strategies [18]. However, systematic evaluations remain scarce regarding its performance under the specific multi-metal contamination profiles and harsh high-altitude conditions typical of Qinghai–Tibet Plateau mining areas. Key unanswered questions include the remediation efficacy of *D. stramonium* in this context, whether patterns exist in the distribution of metals among its root, stem, leaf, and fruit organs, and whether agronomic measures such as planting density can significantly enhance metal removal efficiency.

Based on the aforementioned context, this study focuses on the remediation capacity and mechanisms of the invasive plant *D. stramonium* in toxic metal-contaminated soils from mining areas on the Qinghai–Tibet Plateau. Therefore, we formulated the following hypotheses: (1) higher planting density will significantly improve the total multi-metal removal efficiency from the soil; and (2) *D. stramonium* will exhibit distinct, element-specific organ accumulation and translocation patterns. To test these hypotheses, this study compared the soil removal rates of Pb, Cd, Cr, and As under different planting densities, measured metal concentrations across different plant organs, and calculated the bioconcentration factor (BCF) and translocation factor (TF) to elucidate its remediation pathways (i.e., “stabilization-based” vs. “extraction-based”). Ultimately, by integrating “density regulation” with “accumulation–translocation mechanisms,” this research seeks to propose a quantifiable phytoremediation approach for the ecological restoration of contaminated soils in mining areas on the Qinghai–Tibet Plateau. Importantly, this work evaluates the remediation behavior of an invasive species under strictly controlled experimental conditions, and does not advocate for its unrestricted field deployment without rigorous ecological risk management.

## 2. Materials and Methods

### 2.1. Study Area Overview

The experiment was conducted from 2024 to 2025 at the Key Laboratory of Ecology Park, Tibet University (Lhasa City, Tibet Autonomous Region). The study area, Lhasa, is characterized by a plateau temperate semi-arid monsoon climate. Influenced by topography and monsoon patterns, Lhasa’s climate features abundant sunshine, significant diurnal temperature variation, and distinct wet and dry seasons. According to measurements from the Tibet Meteorological Bureau, Lhasa has an annual average temperature of 9 °C, an annual sunshine duration exceeding 3000 h, and an average annual precipitation of 472 mm. As reported in the Lhasa Municipal People’s Government Gazette, Issue 8 of 2023, the total annual solar radiation in Lhasa is greater than 7.6 × 10^9^ J/m^2^, with an annual average wind speed of 2.69 m/s.

### 2.2. Experimental Materials and Sources of Tested Soils

The tested soil samples were collected from a mining area in Tibet. This mining area is located in the core zone of the copper mining cluster on the Qinghai–Tibet Plateau, with an average elevation of approximately 5200 m, belonging to a typical high-altitude hypoxic environment. Geological background and mining activities in this region have led to varying degrees of combined toxic metal contamination in the local soils.

### 2.3. Experimental Design

Contaminated soil samples were collected from the surface layer (0–20 cm depth) of a mining area in Tibet. The soil was air-dried, followed by the removal of gravel and plant roots, and then sieved for later use. Prior to the pot experiment, the baseline concentrations of toxic metals in the homogenized soil were determined to be 42.1 mg/kg for Pb, 0.26 mg/kg for Cd, 65.4 mg/kg for Cr, and 31.2 mg/kg for As. General physicochemical properties (such as pH, organic matter, and cation exchange capacity) were not fully characterized prior to the experiment, which is recognized as a limitation of the current study. The test plant material consisted of seeds of *D. stramonium.*

A pot experiment was conducted to investigate the remediation effects of different planting densities of *D. stramonium* on the contaminated mining soil. The specific experimental design was as follows:

1. Pot Setup: Uniform plastic pots (upper diameter × height × bottom diameter = 26 cm × 20 cm × 16 cm) were used. Each pot was filled with 5 kg of the sieved contaminated soil.

2. Treatment Groups Based on Planting Density: Three planting density gradients were established:

Low-Density Group (LD): 2 *D. stramonium* plants per pot.

Medium-Density Group (MD): 6 *D. stramonium* plants per pot.

High-Density Group (HD): 10 *D. stramonium* plants per pot.

3. Control Group: An unplanted control group (CK), containing only the contaminated soil, was established.

4. Replication: Initially, each treatment (including the unplanted control) was established with four replicates. However, due to unexpected sample contamination during the preparation and digestion process, only one valid observation was ultimately retained for the unplanted control (CK) during the final soil toxic metal quantification.

5. Randomization and Arrangement: All pots were arranged in a completely randomized design (CRD) to minimize potential edge effects and account for environmental micro-variations. The positions of the pots were rotated regularly during the experiment.

### 2.4. Plant Cultivation and Management

Seeds of *D. stramonium* were germinated and raised in a nursery before being transplanted into the pots. The experiment was conducted over a 136-day period from 19 April 2025, to 2 September 2025. Thinning was performed once the seedlings had developed 4 true leaves, ensuring the final plant count in each pot aligned with the density gradients. During the trial, plants were watered regularly with deionized water every 5 days to maintain normal growth. Pots were equipped with drainage holes at the bottom, and plastic trays were placed underneath to collect any leachate, which was subsequently returned to the pots to prevent the loss of toxic metals. All pots were cultivated under consistent natural light and ambient temperature conditions.

### 2.5. Soil Sample Processing Principles and Procedures

The soil samples were converted into a homogeneous solution through acid digestion using a mixture of nitric acid (HNO_3_), hydrochloric acid (HCl), hydrofluoric acid (HF), and perchloric acid (HClO_4_). This digestate was then nebulized and transported by a carrier gas into the inductively coupled plasma (ICP) torch. Within the high-temperature plasma, the aerosol underwent desolvation, vaporization, dissociation, atomization, and ionization, generating positively charged ions. These ions were extracted into the mass spectrometer via an ion optics system. The mass spectrometer separates ions based on their mass-to-charge ratio (*m*/*z*). For a specific *m*/*z*, the signal intensity is directly proportional to the number of corresponding ions entering the detector, which in turn is proportional to the elemental concentration in the original sample. Thus, elemental concentrations in the sample solution were quantified by measuring the signal intensities obtained by the mass spectrometer.

Exactly 200 mg (weighed to the nearest 0.1 mg) of the air-dried and sieved soil samples were digested using an Aurora 6 microwave digestion system (PreeKem Scientific Instruments Co., Ltd., Shanghai, China) vessel and moistened with a small volume of deionized water. Inside an acid-resistant fume hood, HNO_3_, HCl, and HF were sequentially added to the vessel. The contents were mixed thoroughly to ensure complete contact between the sample and the acids. If a vigorous reaction occurred, the vessel was sealed only after the reaction subsided. The sealed vessel was then placed into the rotor of a microwave digestion system, ensuring proper function of the temperature and pressure sensors. A controlled microwave heating program was executed. Following digestion and subsequent cooling, the vessel was allowed to reach ambient temperature inside the fume hood. Pressure was released cautiously before opening.

The vessel cap was removed, and the contents were heated to evaporate excess acids. The presence of black residues indicated incomplete digestion of organic/carbonaceous material. In such cases, additional aliquots of HNO_3_, HF, and HClO_4_ were added. The mixture was heated at a gentle reflux with the cap loosely on for 30 min, followed by open-vessel heating until dense white fumes of HClO_4_ evolved and the liquid became viscous. This step was repeated if necessary until all black residues were eliminated.

After cooling slightly, the inner walls of the vessel were rinsed with small volumes of dilute HNO_3_ solution using a pipette, utilizing the residual heat to dissolve any adhering residues. The digest was quantitatively transferred into a 25 mL volumetric flask. The rinsing step was repeated, and all washings were combined in the flask. The solution was finally diluted to the mark with dilute HNO_3_, mixed thoroughly, and allowed to stand for 60 min. The supernatant was then taken for analysis. To ensure the analytical quality of soil digestion, procedural blanks and national standard soil reference materials were analyzed concurrently with the samples. The recovery rates for all elements remained within the acceptable range of 95–105%.

### 2.6. Principles and Procedures for Plant Sample Processing

Upon harvest, the plants were separated into roots, stems, leaves, and fruits. To remove adhering soil particles, the roots were thoroughly washed with tap water, followed by three rinses with deionized water. All plant organs were initially oven-dried at 105 °C for 30 min to deactivate enzymes, and subsequently dried at 70 °C to a constant weight. The dried samples were then ground into fine powder.

Exactly 500 mg (weighed to the nearest 0.1 mg) of the ground plant sample was placed into a microwave digestion vessel. After a low-temperature pre-heating step to remove potential gas-producing components, 5 mL of concentrated nitric acid (HNO_3_) was added. Digestion was performed following standard microwave protocols. Upon completion and cooling, the digested solution was degassed, transferred, and diluted to a final volume of 10 mL with deionized water. The elemental concentrations (Pb, Cd, Cr, and As) in the digestates were then determined using inductively coupled plasma mass spectrometry 7800 ICP-MS (Agilent Technologies Inc., Santa Clara, CA, USA). For chemical analysis, strict quality assurance and quality control (QA/QC) procedures were implemented to ensure data reliability. These included the use of procedural blanks, triplicate samples, and certified reference materials (CRMs) for both soil and plant matrices. The recovery rates for the targeted toxic metals (Pb, Cd, Cr, and As) ranged from 95% to 105%, and the relative standard deviation (RSD) of triplicate samples was less than 5%, ensuring the high accuracy and precision of the analytical data.

### 2.7. Data Analysis Methods

1. Toxic Metal Removal Efficiency from Soil: The removal rate for each toxic metal was calculated using Equation (1) [19]:

Removal Efficiency (%) = [(Initial soil metal concentration − Final soil metal concentration)/Initial soil metal concentration] × 100%(1)

2. Bioconcentration Factor (BCF): The BCF, which reflects the plant’s ability to accumulate toxic metals from the soil, was calculated. A BCF value greater than 1 indicates accumulation potential. The BCF was calculated according to previously described methods [7] using Equation (2):

BCF = Metal concentration in a specific plant tissue (dry weight)/Final metal concentration in the corresponding pot’s soil (2)

3. Translocation Factor (TF): The TF, which indicates the plant’s ability to transfer toxic metals from roots to above-ground parts, was calculated. A TF value greater than 1 suggests an effective upward translocation. This factor determines whether the plant acts primarily as a “stabilizer” (by immobilizing metals in roots) or an “extractor” (by transferring metals to above-ground biomass for harvest). To account for the entire above-ground biomass, the TF was calculated following previously described methods [7] using Equation (3):

TF = Average metal concentration in all above-ground parts (stem, leaf, and fruit)/Metal concentration in roots (3)

4. Statistical Analysis: All statistical analyses were performed using R (version 4.2.0, R Core Team). Prior to performing the analysis of variance (ANOVA), the datasets were tested for normality using the Shapiro–Wilk test and for homogeneity of variances using Levene’s test. Significant differences among planting density treatments were determined via one-way ANOVA followed by Tukey’s HSD post hoc test (*p* < 0.05). For treatments with missing or excluded data (e.g., the contaminated replicates in the CK group), the statistical comparisons were handled using a pairwise exclusion approach to maintain analytical integrity. All data visualization and figure generation were conducted using Python (version 3.9) with the Matplotlib (version 3.5.2) and Seaborn (version 0.11.2) libraries.

### 2.8. Biosafety and Waste Management

Given the invasive and toxic nature of *D. stramonium*, the pot experiment was conducted in a strictly controlled area to prevent natural seed dispersal. During the experiment, floral development was closely monitored. Upon completion of the study, all toxic metal-enriched plant biomass (including roots, stems, leaves, and fruits) and residual contaminated soils were carefully collected, sealed in dedicated hazardous waste bags, and transferred to a qualified hazardous waste treatment facility for safe destruction (e.g., high-temperature incineration). These protocols ensured no secondary environmental contamination or biological invasion occurred.

## 3. Results

### 3.1. Toxic Metal Removal Capacity of D. stramonium Under Different Planting Densities

**Figure 1:**
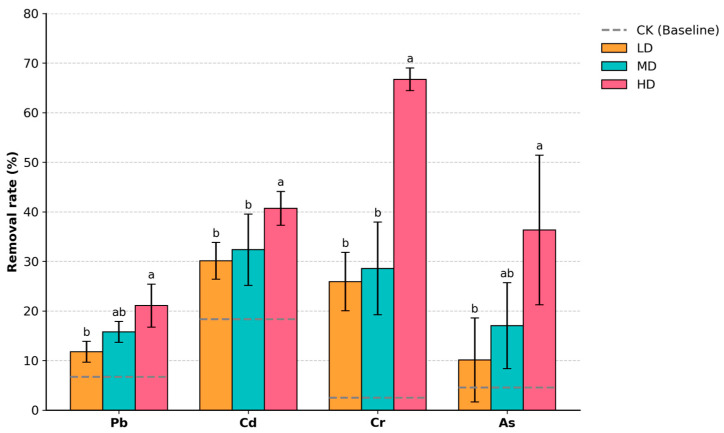
Removal rates of soil toxic metals (Pb, Cd, Cr, and As) under different planting densities of *D. stramonium*. Treatments included low density (LD, 2 plants pot^−1^), medium density (MD, 6 plants pot^−1^), and high density (HD, 10 plants pot^−1^). Bars show mean ± SD, and overlaid dots represent individual replicates. The grey dashed lines represent the baseline reduction observed in the unplanted control (CK) pots for each corresponding metal. Different lowercase letters above the bars indicate statistically significant differences among planting density treatments for the same metal according to one-way ANOVA followed by Tukey’s HSD post hoc test (*p* < 0.05). For each metal, bars sharing the same letter are not significantly different, while bars with different letters (e.g., ‘a’ vs. ‘b’) indicate a statistically significant difference.

Planting density significantly influenced the removal rates of soil toxic metals by *D. stramonium* (Figure 1). Compared to the unplanted control (CK), all planting treatments enhanced the removal of Pb, Cd, Cr, and As, with a general trend of increased removal efficiency corresponding to higher planting densities. The Pb removal rate rose from 6.7% in the CK to 11.8% in the LD (2 plants/pot), 15.8% in the MD (6 plants/pot), and 21.1% in the HD (10 plants/pot) treatments. Similarly, the Cd removal rate increased with density, recording 18.4% (CK), 30.1% (LD), 32.4% (MD), and 40.7% (HD). The Cr removal was only 2.5% in the CK but improved to approximately 25.9–28.6% in the low- and medium-density treatments. The high-density treatment further increased the removal significantly to 66.7%. The As removal rate showed greater variability but generally increased with planting density: 4.6% (CK), 10.1% (LD), 17.0% (MD), and a maximum of 36.3% (HD). Overall, these results indicate that increasing the planting density can enhance the soil toxic metal removal capacity of *D. stramonium* for multiple metals, with the most pronounced improvement observed for Cr.

As shown in Table S1, different planting densities significantly affected the toxic metal removal rates from soil by *D. stramonium*. For Pb, the removal rate showed no significant difference between the CK and LD treatments (both labeled ‘b’), while the MD and HD treatments were significantly higher than CK/LD (labeled ‘a’), with the HD treatment achieving the highest value (21.1%). The Cd removal rate was significantly lowest in the CK (labeled ‘b’), whereas the LD, MD, and HD treatments were all significantly higher than CK (all labeled ‘a’), with the HD treatment reaching 40.71%. The density effect was most pronounced for Cr: CK, LD, and MD were all in group ‘b’, while the HD treatment increased significantly to group ‘a’, with an average removal rate of 66.7%. The As removal rate was lowest in the LD treatment (labeled ‘b’), which was significantly lower than the CK, MD, and HD treatments (all labeled ‘a’), and generally increased with planting density, reaching 36.3% in the HD treatment. Overall, the results indicate that increasing planting density can enhance the removal capacity for Pb, Cd, and Cr, with the most significant improvement observed for Cr.

### 3.2. Concentrations and Enrichment Capacity of Four Soil Toxic Metals in Different Organs of D. stramonium

#### 3.2.1. Concentrations of Four Toxic Metals in Different Organs of *D. stramonium*

**Figure 2:**
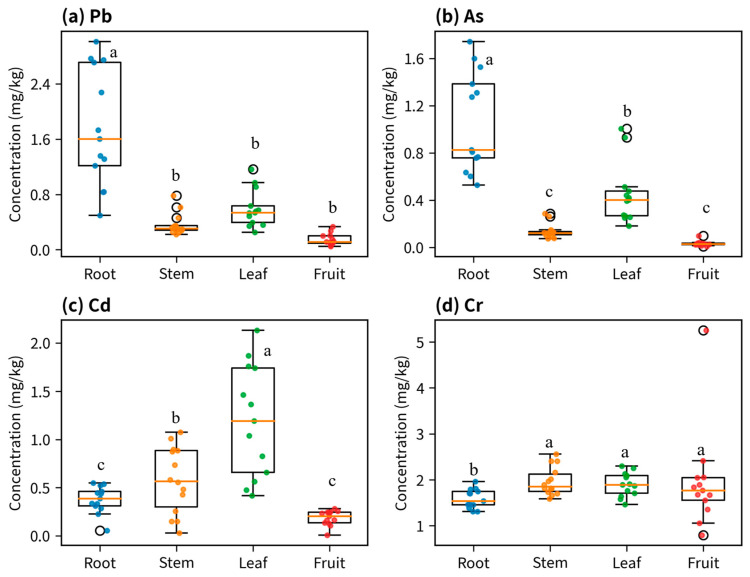
Concentrations of four toxic metals (Pb, As, Cd and Cr) in different organs of *D. stramonium* (root, stem, leaf and fruit). Bars represent mean ± standard deviation (SD), and overlaid dots indicate individual replicate measurements. Units are mg/kg. Note: Data represent the pooled values across all planting density treatments. Boxes within the same subfigure labeled with different lowercase letters (a, b, c) represent statistically significant differences (*p* < 0.05) among plant organs for that specific metal, according to Tukey’s HSD test.

The results revealed distinct differences in the concentrations of the four toxic metals among different organs of *D. stramonium* (data were pooled across all planting density treatments to illustrate general physiological allocation patterns; Figure 2). Pb and As were primarily enriched in the roots, with root concentrations being significantly higher than those in stems, leaves, and fruits (Tukey’s HSD, *p* < 0.05). The lowest concentrations for both metals were found in stems (Pb 0.36 ± 0.16; As 0.14 ± 0.06), while fruits showed the lowest As content (0.034 ± 0.021). The distribution pattern of Cd differed from that of Pb/As. The highest Cd concentration was observed in leaves (1.19 ± 0.58), followed by stems (0.58 ± 0.34) and roots (0.38 ± 0.14), with fruits containing the lowest level (0.19 ± 0.08). Cr concentrations showed relatively smaller variations among organs, with mean values ranging approximately from 1.59 to 1.97 mg/kg. However, fruit Cr content exhibited substantial variance with notable high-value outliers, suggesting pronounced inter-replicate variability in Cr accumulation within reproductive organs. Overall, roots demonstrated a preference for enriching Pb and As, and leaves for Cd, while Cr distribution was more balanced among organs, albeit with higher variability in fruits.

#### 3.2.2. Enrichment Capacity of *D. stramonium* for Soil Toxic Metals in Different Organs

**Figure 3:**
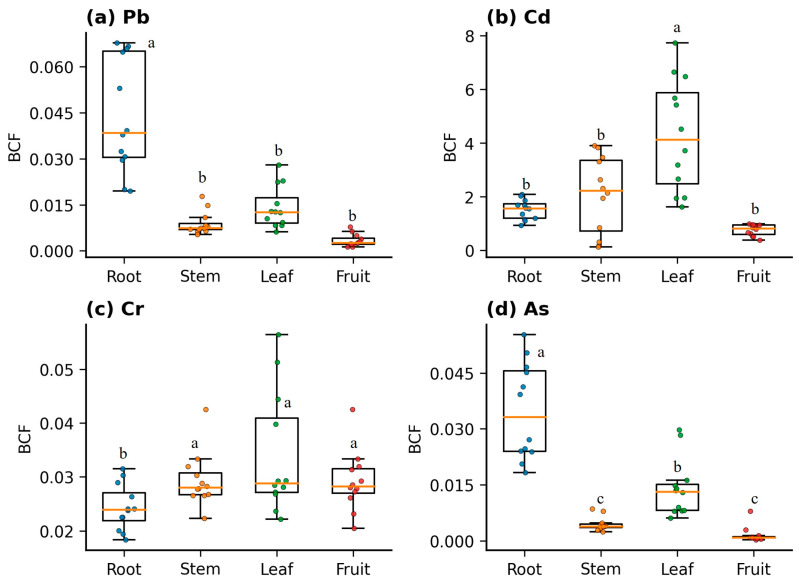
Bioconcentration factors (BCF) of toxic metals (Pb, Cd, Cr, and As) in different organs (root, stem, leaf, and fruit) of *D. stramonium*. Boxplots represent the median, interquartile range, and whiskers (1.5 × IQR), while overlaid colored dots indicate individual replicates. BCF values were calculated as the ratio of the metal concentration in each plant organ to the final metal concentration in the corresponding soil (Equation (2)). Different lowercase letters above the boxes indicate statistically significant differences among plant organs for each toxic metal based on one-way ANOVA followed by Tukey’s HSD post hoc test (*p* < 0.05). Note: Data are pooled across all planting density treatments. Boxes within the same subfigure labeled with different lowercase letters (a, b, c) represent statistically significant differences (*p* < 0.05) among plant organs for that specific metal, according to Tukey’s HSD test.

Based on the BCF (Bioconcentration Factor, BCF = metal concentration in plant organ/metal concentration in soil) data (Figure 3), *D. stramonium* exhibited distinct organ-specific and element-specific patterns in the enrichment of different toxic metals. Overall, the magnitudes and distribution patterns of BCF varied among metals, indicating differences in the absorption, translocation, and allocation mechanisms of *D. stramonium* for these elements.

For Pb, the root BCF was significantly higher than in other organs. Both scatter and box plots showed a markedly higher median and upper quartile for roots, with relatively greater individual variation. Leaves displayed intermediate values, while stems and fruits generally had lower BCF. The root BCF for Pb mostly ranged between 0.02 and 0.07, whereas stems ranged between 0.005 and 0.02, and fruits between 0.001 and 0.008. This suggests that Pb is primarily enriched in the below-ground parts, with limited translocation to above-ground tissues. Cd exhibited the highest enrichment capacity and the most pronounced organ-specific differences. Leaf BCF for Cd was significantly the highest, with a wide data distribution (approximately 1.6–7.7). The median and upper quartile for leaves were markedly higher than for roots, stems, and fruits, indicating a strong tendency for Cd accumulation in above-ground parts. Stem BCF was also relatively high (approximately 0.1–3.9) but generally lower than leaves. Roots showed intermediate levels (approximately 0.9–2.1), while fruits had the lowest BCF (approximately 0.38–0.99). This pattern indicates that Cd is readily translocated to and accumulated in leaves, with restricted allocation to fruits.

The BCF values for Cr were generally low across all organs, with relatively small differences among them, mostly concentrated between 0.018 and 0.056. The distributions for roots, stems, and fruits were similar, while leaves were slightly higher with somewhat greater variability. This indicates a relatively balanced distribution of Cr within the plant, though leaves may still be a relatively more enrichment-prone organ. Compared to Pb and Cd, the differentiation of Cr among organs was less distinct.

For As, the root BCF was the highest and relatively concentrated (approximately 0.018–0.056), followed by leaves (approximately 0.06–0.03). Stem BCF was significantly lower (approximately 0.002–0.01), and fruits had the lowest values (mostly between 0.0003 and 0.003, with occasional relatively higher points in individual samples). This indicates that As is primarily enriched in roots, with restricted translocation to stems and fruits. Although leaves can accumulate a certain proportion, their BCF remains significantly lower than roots. Although leaves can accumulate a certain proportion, their BCF remains significantly lower than roots.

In summary, the enrichment characteristics of *D. stramonium* can be described as follows: roots show a greater tendency to enrich Pb and As; leaves are the primary enrichment organ for Cd; and Cr is relatively evenly distributed among organs, though slightly higher in leaves. These findings provide a data basis for subsequent evaluation of the phytoremediation potential of *D. stramonium* in toxic metal-contaminated soils, as well as for risk management and post-harvest disposal strategies for different plant organs.

One-way analysis of variance (ANOVA) indicated (Table S2) that significant differences existed in the BCF values among different organs (roots, stems, leaves, fruits) of *D. stramonium* for the same toxic metal (Pb: F = 38.18, *p* < 0.001; Cd: F = 17.03, *p* < 0.001; Cr: F = 3.65, *p* = 0.02; As: F = 45.71, *p* < 0.001). Post hoc Tukey HSD tests further revealed that for Pb, the BCF was highest in the roots (0.04 ± 0.02, ^a^), significantly greater than in stems, leaves, and fruits (all ^b^), indicating primary Pb enrichment in the roots. For Cd, the BCF was highest in the leaves (4.30 ± 2.08, ^a^), significantly higher than in roots, stems, and fruits (all ^b^), suggesting Cd is more readily translocated to and accumulated in the shoots. Organ differences for Cr were relatively small; however, leaves, stems, and fruits (all ^a^) showed significantly higher BCF than roots (0.02 ± 0.01, ^b^), indicating a slight tendency for preferential Cr accumulation in above-ground tissues. For As, a gradient of root > leaf > stem ≈ fruit was observed. The root BCF was the highest (0.035 ± 0.01, ^a^), followed by leaves (0.01 ± 0.0077, ^b^), with stems and fruits showing the lowest and statistically similar values (0.0045 ± 0.0019 and 0.0016 ± 0.0021, respectively, both ^c^). Overall, roots exhibited a preference for enriching Pb and As, and leaves were the primary enrichment organ for Cd, while Cr showed weaker inter-organ variation with a marginally higher accumulation in above-ground parts.

### 3.3. Translocation Capacity of Toxic Metals in D. stramonium

**Figure 4:**
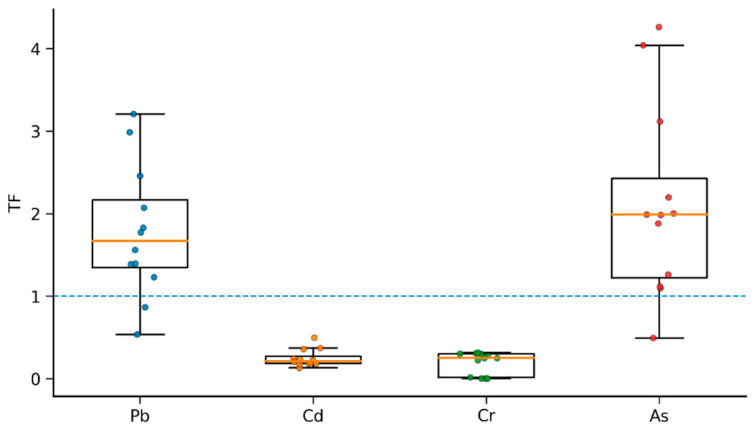
Translocation factors (TF) of toxic metals (Pb, Cd, Cr and As) in *D. stramonium*. Boxplots show the median, interquartile range and whiskers (1.5 × IQR), and overlaid colored dots represent individual replicates. The dashed horizontal line indicates TF = 1 (equal distribution between below- and above-ground parts).

Based on the translocation factor (TF) data, distinct differences were observed in the translocation capacity of different toxic metals within *D. stramonium* (Figure 4). Overall, TF values for As and Pb were predominantly greater than 1, while those for Cd and Cr were consistently less than 1.

Specifically, the TF for Pb varied within a range of approximately 0.54–3.21, with most samples exceeding 1, indicating a certain capacity for Pb translocation to the aerial parts, albeit with considerable inter-individual variation. The TF for As ranged from approximately 0.49 to 4.26, with the majority of samples being significantly higher than 1, suggesting that As is more readily translocated and accumulated in the aerial parts, demonstrating the strongest translocation capacity among the metals studied.

In contrast, the TF for Cd remained consistently below 1 (approximately 0.13–0.50), indicating that Cd is primarily retained in the belowground parts, with limited translocation to the shoots. Similarly, the TF for Cr was also generally below 1 (approximately 0.002–0.315), with the presence of very low values (~0.002–0.003), indicating extremely low root-to-shoot translocation efficiency.

In summary, *D. stramonium* exhibits a relatively strong capacity for translocating As and Pb to its aerial parts, while its translocation capacity for Cd and Cr is weaker, reflecting clear element-specific translocation patterns.

## 4. Discussion

This study systematically investigated the phytoremediation performance of *D. stramonium* in multi-metal-contaminated soil, focusing on comparing soil removal rates under different planting densities and the differences in toxic metal concentrations, bioconcentration factors (BCF), and translocation factors (TF) across various plant organs. Overall, increasing planting density significantly enhanced the removal efficiency at the population level. Furthermore, the metals exhibited distinct element-specific patterns of “organ allocation—enrichment/translocation”: Pb and As were preferentially enriched in the roots, Cd showed the strongest accumulation in leaves, while Cr demonstrated low translocation and its removal rate was most sensitive to changes in planting density. These patterns collectively suggest that *D. stramonium* exhibits element-specific remediation strategies: it shows partial aboveground accumulation for Cd, rhizosphere immobilization and belowground enrichment for Pb/As, and relies more on rhizosphere process-driven removal and stabilization for Cr [20,21].

The removal rate results showed that the high-density (HD) treatment generally outperformed the medium-density (MD) and low-density (LD) treatments, with the most pronounced enhancement observed for Cr. Increased planting density has been reported to improve phytoremediation efficiency through two primary pathways: first, by increasing the total biomass and root length per unit area/pot, thereby enhancing the “sink strength” for metal uptake; and second, by intensifying rhizospheric processes (root respiration, exudation, and microbial interactions), which can alter metal speciation and bioavailability, consequently increasing the bioavailable fraction [21]. Although we did not directly measure specific root traits, root exudation, or microbial communities in this study, we hypothesize that these intensified rhizospheric factors may have contributed to the observed density effects.

In multi-metal scenarios, the magnitude of response to density varies among different metals. Existing field studies indicate that planting density and harvest strategies significantly influence the removal efficiency and system sustainability at multi-metal-contaminated sites. Notably, higher density is not always optimal for all metals, often necessitating a trade-off between efficiency and maintainability by integrating biomass management and harvest strategies [20].

Therefore, the amplified effect on Cr removal observed in the HD group might not solely be attributed to increased biomass. Although we did not directly evaluate soil chemical speciation in this study, we hypothesize that this enhancement could theoretically be associated with rhizosphere environmental changes promoting the transformation/complexation of Cr (VI) and its subsequent adsorption/immobilization. Previous studies have demonstrated that exogenous chelating agents (e.g., EDTA, oxalic acid) significantly enhance Cr(VI) phytoextraction by *D. stramonium*, further supporting the premise that effective Cr remediation relies heavily on modulating its soil chemical speciation [18]. It is also important to address the background reduction of toxic metals observed in the unplanted control (CK) pots, which varied significantly among elements (e.g., 18.36% for Cd vs. 2.48% for Cr). Since no plants were present to extract the metals, this baseline attenuation can be attributed to abiotic experimental factors over the 136-day cultivation period. First, despite the use of bottom trays to return leachate, the inherent high mobility and solubility of certain elements in soils—particularly Cd—make them susceptible to minor leaching or downward spatial redistribution within the soil profile during repeated watering cycles. In contrast, elements like Cr and Pb are typically strongly adsorbed to soil colloids and form insoluble precipitates, thereby exhibiting much lower background losses. Second, potential long-term adsorption of free metal ions onto the inner walls of the plastic pots may also contribute to the apparent decrease in extractable soil concentrations. Regardless of these abiotic background losses, the overall removal efficiencies in all planted treatments were substantially higher than their respective CK baselines, confirming the active and primary role of *D. stramonium* in the phytoremediation system.

The concentrations of toxic metals in roots, stems, leaves, and fruits (Table S5), along with the BCF results (Table S3), consistently reveal that *D. stramonium* employs differentiated allocation strategies for different metals. BCF and TF are two of the most commonly used indicators for evaluating phytoremediation potential: BCF reflects the plant’s enrichment capacity relative to the growth medium (soil or solution), while TF indicates the translocation capacity from roots to shoots [22,23]. For Pb and As, BCF values were significantly higher in roots than in other organs, as supported by ANOVA and Tukey’s test, indicating that these metals are primarily enriched and immobilized in belowground tissues. This observation aligns with the widely observed phenomenon that Pb tends to bind to root cell walls and extracellular matrices, leading to its sequestration in roots and restricted long-distance transport [24]. In the case of As, although roots remain the main enrichment site, the higher BCF in leaves compared to stems and fruits suggests a certain capacity for As redistribution to aboveground parts, a finding further supported by subsequent TF results. The uptake and translocation of As in plants are closely linked to its chemical speciation: arsenate As(V) can enter via phosphate transport systems and participate in xylem loading, whereas arsenite As (III) is often associated with NIP-type aquaporins [25,26]. Consequently, under varying soil phosphorus levels and As speciation, the organ distribution and translocation of As exhibit greater plasticity and variability [27].

The significantly highest BCF for Cd in the leaves indicates a localized tendency to accumulate in aboveground tissues. However, as our overall TF for Cd remained below 1, this indicates that the lower Cd concentrations in stems and fruits dilute the overall aboveground burden. Thus, while partial removal can be achieved via leaf harvesting, the plant does not function as a highly efficient whole-shoot extractor for Cd. After entering the roots, Cd can be loaded into the xylem via various metal transporters and transported over long distances to the leaves through the transpiration stream. Among these transporters, toxic metal ATPases such as HMA2/HMA4 are considered key players in root-to-shoot translocation [28,29]. Experiments on *D. stramonium* have also reported its potential as a Cd-accumulating plant, with enhancement of remediation performance achievable through measures such as chitosan/chitosan nanoparticles (literature indicates TF values >1 under certain conditions) [17]. Although the TF data in this study show Cd overall <1 (indicating restricted translocation), the BCF reveals significant enrichment in leaves. This suggests that under the experimental soil conditions and metal speciation background, the “root uptake–shoot allocation” of Cd may be environmentally constrained by factors such as soil available fractions, competing ions, rhizosphere pH, etc., resulting in a relatively low TF while leaves still serve as the main accumulation organ. This discrepancy underscores that the TF/BCF of a given plant are not fixed attributes but rather the outcome of the complex interaction among plant genotype, soil chemistry, and cultivation management [21,23].

Regarding Cr, the BCF values were generally low with relatively small differences among organs. However, combined with the TF results (overall significantly below 1, with notably low values observed), it can be concluded that Cr tends to be immobilized in the roots, with limited translocation to the aboveground parts. Numerous studies have indicated that Cr lacks specific transport systems in plants, often entering via non-specific channels such as sulfate or iron transporters, and primarily accumulates in root tissues with restricted translocation to shoots [18,26]. Therefore, the significant response of Cr removal rate to high planting density observed in this study likely reflects enhanced absorption/immobilization due to increased root system scale and intensified rhizosphere processes, rather than efficient aboveground extraction.

The TF results (Table S4) clearly show that most samples of As and Pb exceeded 1, indicating a certain degree of root-to-shoot translocation capacity, with As exhibiting a broader fluctuation range; both Cd and Cr were significantly below 1, reflecting retention in belowground parts or restricted translocation. This pattern is closely associated with the detoxification/sequestration strategies of metals in plants. Pb is generally considered a “low-mobility” metal in most plants, where toxicity can be reduced through mechanisms such as cell wall binding and intracellular vacuolar sequestration in roots, often resulting in a pattern of “root enrichment with restricted shoot translocation” [26]. In contrast, due to its coupling with phosphorus metabolism and the transmembrane transport characteristics of As (III), As may be more prone to long-distance transport under varying environmental conditions, particularly when plants absorb As(V) via phosphate transport systems, potentially leading to increased accumulation in aboveground tissues [25,27]. For Cd, a TF < 1 suggests limited loading to shoots or enhanced sequestration in roots; while the extremely low TF for Cr aligns well with literature reports that “Cr predominantly remains in roots with very limited translocation to stems and leaves” [30,31].

From an engineering application perspective, TF and organ-specific enrichment determine harvesting and disposal strategies: if the goal is “removal,” metals should be enriched in harvestable aboveground parts as much as possible; if the goal is “stabilization/risk control,” root immobilization is also meaningful, but precautions must be taken to prevent re-release upon root decay. For *D. stramonium*, the prominent enrichment of Cd in leaves implies that partial removal can be achieved through aboveground harvesting; the stronger root enrichment of Pb/As suggests a more significant rhizosphere immobilization effect, which may be better suited for strategies involving root–soil matrix removal or stabilization management; Cr, however, requires a combination with soil amendments (e.g., speciation transformation, complexation/reduction) to enhance its removable fraction [18,21,31].

Although higher planting density improved removal rates, practical applications must also consider issues such as growth inhibition per plant due to competition, increased lodging risk, greater demand for nutrients and water, and higher harvesting costs. Field studies indicate that the combination of planting density and harvesting methods affects system sustainability, and higher density is not always optimal [20]. Furthermore, phytoremediation often faces the bottleneck of contaminated biomass disposal: plant residues enriched with metals can cause secondary contamination if not handled properly. Common disposal methods include pyrolysis, incineration, compaction, and secure landfilling, all of which involve cost or safety constraints [21]. It is worth noting that *D. stramonium* itself contains toxic alkaloids, which, while reducing the direct risk of entering the food chain, also necessitate strict management of the post-remediation biomass to prevent uncontrolled disposal or use as fodder. More importantly, we explicitly emphasize that our findings only support the remediation evaluation of this invasive species under strictly controlled experimental conditions; we do not advocate for its straightforward or unrestricted field deployment, which could pose severe ecological risks.

Based on pot experiments with density gradients and organ-specific distribution data, this study has revealed the differential enrichment and translocation patterns of *D. stramonium* for different metals. However, several key questions warrant further investigation: First, isotopic or speciation analysis should be integrated to clarify changes in the bioavailable fractions of Pb, Cd, Cr, and As in the soil and the underlying rhizospheric mechanisms, which could explain why Cr responded most strongly to planting density. Second, a limitation of the current study is that we primarily focused on tissue metal concentrations and did not systematically record the final dry biomass of separated organs across all density treatments. Because total metal phytoextraction is fundamentally a function of both tissue concentration and biomass yield, future studies must incorporate precise biomass measurements to calculate the absolute mass balance and total extraction efficiencies under varying planting densities. Third, under safe preconditions, exploring the synergistic effects of soil amendments (e.g., organic acids, chelating agents, microbial enhancement) with density-based planting is recommended, particularly for low-mobility metals such as Cr and Pb [18,21,32].

In summary, *D. stramonium* demonstrates strong adaptability and distinct element-specific remediation potential in multi-metal contaminated soils. Increasing planting density can enhance overall removal efficiency. Cd is more likely to be extracted via aboveground (leaf) enrichment, while Pb and As tend to be enriched and immobilized in the roots. Cr is characterized by low translocation and rhizosphere process-driven removal/stabilization. Furthermore, while pot experiments provide valuable baseline data, future field-scale implementation must carefully evaluate the economic feasibility of specialized biomass disposal facilities against the remediation benefits. Ultimately, practical implementation hinges on the development of optimal planting density combined with tailored harvesting and strict disposal protocols to balance remediation efficacy with ecological safety.

## 5. Conclusions

This study demonstrates that the invasive plant *D. stramonium* possesses notable remediation potential for toxic metal-contaminated soils in mining areas of the Qinghai–Tibet Plateau, with planting density being a key factor influencing remediation efficiency. Compared to the unplanted control, cultivation significantly enhanced the soil removal rates of Pb, Cd, Cr, and As, with a general trend of increasing removal corresponding to higher planting densities. The high-density treatment achieved the highest removal performance, with Cr showing the most pronounced response. Furthermore, toxic metals exhibited distinct organ-specific allocation patterns within the plant: Pb and As were primarily enriched in the roots, Cd accumulation was most prominent in the leaves, while overall enrichment and translocation of Cr were relatively low. Integrated analysis of the BCF and TF indicates that *D. stramonium* exhibits greater potential for “extraction-based” remediation of Cd through aboveground accumulation, whereas it displays “stabilization-based” characteristics for Pb and As via root enrichment and immobilization. Although Cr showed low translocation, its removal increased significantly under high planting density. These findings suggest that practical applications should optimize planting density and harvesting strategies according to target metal types, ensure safe disposal of contaminated biomass, and strengthen risk management to control potential invasive spread.

## Data Availability

The original contributions presented in this study are included in the article/Supplementary Material. Further inquiries can be directed to the corresponding author(s).

## Supplementary Materials

The following supporting information can be downloaded at https://www.mdpi.com/article/10.3390/biology15100807/s1. Table S1. Removal rates (%) of soil heavy metals (Pb, Cd, Cr and As) by *D. stramonium* under different planting densities with Tukey HSD grouping letters (*p* < 0.05). Table S2. One-way ANOVA and Tukey’s HSD post-hoc test for bioconcentration factors (BCF; mean ± SD) of heavy metals (Pb, Cd, Cr and As) in different organs (root, stem, leaf and fruit) of *D. stramonium.* Table S3. Bioconcentration factors (BCF) of heavy metals in different organs of *D. stramonium* separated by individual planting density treatments. Table S4. Translocation factors (TF) of heavy metals in *D. stramonium* separated by individual planting density treatments. Table S5. Concentrations of heavy metals in different organs of *D. stramonium* separated by individual planting density treatments.

## References

[1] Feng S., Li Z., Zhang C., Qi R., Yang L. (2025). Ecological restoration in high-altitude mining areas: Evaluation of soil reconstruction and vegetation recovery in the Jiangcang coal mining area on the Qinghai–Tibet Plateau. Front. Environ. Sci..

[2] Qi J., Lu X., Sai N., Liu Y., Du W. (2024). Heavy metal concentrations in soil and ecological risk assessment of mining areas in the Qinghai–Tibet Plateau. PeerJ.

[3] Du H., Wang J., Wang Y., Yao Y., Liu X., Zhou Y. (2023). Contamination characteristics, source analysis, and spatial prediction of heavy metals in soils on the Qinghai–Tibetan Plateau. J. Soils Sediments.

[4] Wei P., Shao T., Wang R., Chen Z., Zhang Z., Xu Z., Zhu Y., Li D., Fu L., Wang F. (2020). A Study on Heavy Metals in the Surface Soil of the Region around the Qinghai Lake in Tibet Plateau: Pollution Risk Evaluation and Pollution Source Analysis. Water.

[5] Ye L., Xiang M., Zhou H., Gao X., Liu X., Wang Y., Wu J. (2026). Ecological risk assessment and source apportionment of soil heavy metal contamination in a typical alpine mining area on the Qinghai–Tibet Plateau. Environ. Manag..

[6] Ali H., Khan E., Sajad M.A. (2013). Phytoremediation of heavy metals—Concepts and applications. Chemosphere.

[7] Salt D.E., Blaylock M., Kumar P.B.A.N., Dushenkov V., Ensley B.D., Chet I., Raskin I. (1995). Phytoremediation: A novel strategy for the removal of toxic metals from the environment using plants. Nat. Biotechnol..

[8] Zha X., An J., Deng L., Gao X., Tian Y. (2024). Risk assessment and source apportionment of heavy metals in the soil–water–grain system in a typical area of the central Qinghai–Tibet Plateau. Ecol. Indic..

[9] Raskin I., Smith R.D., Salt D.E. (1997). Phytoremediation of metals: Using plants to remove pollutants from the environment. Curr. Opin. Biotechnol..

[10] Salt D.E., Smith R.D., Raskin I. (1998). Phytoremediation. Annu. Rev. Plant Physiol. Plant Mol. Biol..

[11] Deepika, Tyagi A., Haritash A.K. (2025). Environmental impacts of mine tailings and phytoremediation as a sustainable management strategy: A review. Acta Geochim..

[12] Xu H., Qiao X., Gao G., Li Y., Zhang Z., Liu J. (2026). Advances in ecological restoration of mining-impacted landscapes: Techniques, case studies, and key challenges. Environ. Res..

[13] Chu Q., Liu Y., Peng C., Zhang Y., Cernava T., Qiong L., Zhou Y., Siddiqui J.A., Ghani M.I., Wang Q. (2024). Invasive alien plants in the Qinghai-Tibetan Plateau (China): Current state and future predictions. Ecol. Indic..

[14] Wang J.W., Chen Y.H., Xu M., Chen J. (2023). Prediction of potential risk area of the invasive plant *Datura stramonium* L. in Tibet under the background of climate change. Acta Ecol. Sin..

[15] Wei K., Guo T. (2023). The Changes of Tolerance, Accumulation and Oxidative Stress Response to Cadmium in Tobacco Caused by Introducing *Datura stramonium* L. Genes. Agronomy.

[16] Chen Y., Zeng Z., La Q., Wang J. (2025). Invasion Mechanisms of the Alien Plant *Datura stramonium* in Xizang: Insights from Genetic Differentiation, Allelopathy, and Ecological Niche Analysis. Biology.

[17] Shirkhani Z., Chehregani Rad A., Mohsenzadeh F. (2021). Improving Cd-phytoremediation ability of *Datura stramonium* L. by chitosan and chitosan nanoparticles. Biologia.

[18] Shi C., Lv J., Pei Z., Wang H., Chang N., Fang X., Wang K. (2024). Study on the enhancement effect of EDTA and oxalic acid on phytoremediation of Cr (VI) from soil using *Datura stramonium* L. Ecotoxicol. Environ. Saf..

[19] Sarkodie E.K., Li K., Guo Z., Yang J., Deng Y., Shi J., Peng Y., Jiang Y., Jiang H., Liu H. (2025). The Effect of Cysteine on the Removal of Cadmium in Paddy Soil by Combination with Bioremediation and the Response of the Soil Microbial Community. Toxics.

[20] Luo J., He M., Qi S.H., Wu J.S., Gu X.S. (2018). Effect of planting density and harvest protocol on field-scale phytoremediation efficiency by *Eucalyptus globulus*. Environ. Sci. Pollut. Res..

[21] Shen X., Dai M., Yang J., Sun L., Tan X., Peng C., Ali I., Naz I. (2022). A critical review on the phytoremediation of heavy metals from environment: Performance and challenges. Chemosphere.

[22] Vračko M. (2001). ScienceDirect Topics. Bioconcentration Factor (BCF)—An Overview.

[23] Usman K., Al Jabri H., Abu-Dieyeh M.H., Al-Ghouti M.A. (2020). Comparative Assessment of Toxic Metals Bioaccumulation and the Mechanisms of Chromium (Cr) Tolerance and Uptake in *Calotropis procera*. Front. Plant Sci..

[24] Collin S., Baskar A., Geevarghese D.M., Ali M.N.V.S., Bahubali P., Choudhary R., Lvov V., Tovar G.I., Senatov F., Koppala S. (2022). Bioaccumulation of lead (Pb) and its effects in plants: A review. J. Hazard. Mater. Lett..

[25] Mendoza-Cózatl D.G., Jobe T.O., Hauser F., Schroeder J.I. (2011). Long-distance transport, vacuolar sequestration, tolerance, and transcriptional responses induced by cadmium and arsenic. Curr. Opin. Plant Biol..

[26] Li N., Wang J., Song W.-Y. (2016). Arsenic Uptake and Translocation in Plants. Plant Cell Physiol..

[27] Khan A., Kanwal F., Shahzad M., Naz S., Jalil S., Zhang G. (2025). Interactions of arsenic and phosphorus in their uptake and transportation in plants: Advances and prospective research on the mechanisms and approaches for alleviating arsenic stress. J. Integr. Agric..

[28] Jalmi S.K., Kumar K., Srivastava S. (2022). The Role of ABC Transporters in Metal Transport in Plants. Plant Metal and Metalloid Transporters.

[29] Tao J., Lu L. (2022). Advances in Genes-Encoding Transporters for Cadmium Uptake, Translocation, and Accumulation in Plants. Toxics.

[30] Shahid M., Shamshad S., Rafiq M., Khalid S., Bibi I., Niazi N.K., Dumat C., Rashid M.I. (2017). Chromium speciation, bioavailability, uptake, toxicity and detoxification in soil–plant system: A review. Chemosphere.

[31] Sharma A., Kapoor D., Wang J., Shahzad B., Kumar V., Bali A.S., Jasrotia S., Zheng B. (2020). Chromium in plants: Uptake, transport, physiological effects and recent advances in molecular investigations. Ecotoxicol. Environ. Saf..

[32] Fahr M., Laplaze L., Bendaou N., Hocher V., Mzibri M.E., Bogusz D., Smouni A. (2013). Effect of lead on root growth. Front. Plant Sci..

