# Supplementary material for: Phytoremediation Potential of the Invasive Plant Datura stramonium (Solanaceae) for Toxic Metal Removal from Soil in the Qinghai–Tibet Plateau
Source: Biology (Basel). 2026 May 19;15(10):807. doi: 10.3390/biology15100807 (PMC13203727; doi:10.3390/biology15100807)
Supplement: Supplementary file 1 [file biology-15-00807-s001.zip › Supplementary_Materials.pdf]

Table S1. Removal rates (%) of soil heavy metals (Pb, Cd, Cr and As) by *D. stramonium* under different planting densities with Tukey HSD grouping letters (P < 0.05).

Treatment	Pb removal rate (%)	Cd removal rate (%)	Cr removal rate (%)	As removal rate (%)
CK	6.73	18.36	2.48	4.57
LD	11.79 ± 2.10 (b)	30.12 ± 3.73 (b)	25.91 ± 5.89 (b)	10.14 ± 8.47 (b)
MD	15.78 ± 2.12 (ab)	32.36 ± 7.20 (b)	28.59 ± 9.35 (b)	17.04 ± 8.68 (ab)
HD	21.08 ± 4.33 (a)	40.71 ± 3.40 (a)	66.73 ± 2.27 (a)	36.33 ± 15.08 (a)

note: Data are presented as mean ± standard deviation (SD) for the heavy metal removal rate (%). CK denotes the unplanted control; LD, MD, and HD denote low density (2 plants pot^{-1}), medium density (6 plants pot^{-1}), and high density (10 plants pot^{-1}), respectively. Within the same column, different lowercase letters indicate significant differences among treatments (Tukey's HSD, $p < 0.05$). The CK group was excluded from the ANOVA due to the retention of only one valid observation after unexpected contamination; its data are presented solely as a baseline to indicate natural background attenuation.

Table S2. One-way ANOVA and Tukey's HSD post-hoc test for bioconcentration factors (BCF; mean ± SD) of heavy metals (Pb, Cd, Cr and As) in different organs (root, stem, leaf and fruit) of *D. stramonium.*

HM	Root (BCF)	Stem (BCF)	Leaf (BCF)	Fruit (BCF)	F	p
Pb	0.0440 ± 0.0187 (a)	0.0089 ± 0.0037 (b)	0.0141 ± 0.0068 (b)	0.0034 ± 0.0020 (b)	38.177	2.614e-12
Cd	1.5210 ± 0.3724 (b)	2.0878 ± 1.4238 (b)	4.3042 ± 2.0841 (a)	0.7577 ± 0.2074 (b)	17.030	1.742e-07
Cr	0.0244 ± 0.0042 (b)	0.0294 ± 0.0050 (a)	0.0339 ± 0.0113 (a)	0.0292 ± 0.0055 (a)	3.645	0.01967
As	0.0347 ± 0.0130 (a)	0.0045 ± 0.0019 (c)	0.0140 ± 0.0077 (b)	0.0016 ± 0.0021 (c)	45.707	1.425e-13

Note: Different lowercase letters within the same row indicate significant differences among plant organs (Tukey's HSD test, *P* < 0.05), while the same letter indicates no significant difference. BCF = metal concentration in plant organ / metal concentration in soil.

Table S3. Bioconcentration factors (BCF) of heavy metals in different organs of *D. stramonium* separated by individual planting density treatments.

Heavy Metal	Organ	LD (Mean ± SD)	MD (Mean ± SD)	HD (Mean ± SD)
**Pb**	Root	0.035 ± 0.005	0.055 ± 0.023	0.042 ± 0.021
	Stem	0.010 ± 0.006	0.009 ± 0.004	0.008 ± 0.002
	Leaf	0.009 ± 0.003	0.020 ± 0.007	0.014 ± 0.006
	Fruit	0.004 ± 0.003	0.003 ± 0.002	0.004 ± 0.002
**Cd**	Root	1.603 ± 0.553	1.478 ± 0.257	1.483 ± 0.351
	Stem	2.347 ± 1.691	2.751 ± 0.794	1.165 ± 1.464
	Leaf	4.242 ± 2.281	5.023 ± 2.046	3.648 ± 2.295
	Fruit	0.805 ± 0.197	0.610 ± 0.213	0.858 ± 0.167
**Cr**	Root	0.028 ± 0.005	0.023 ± 0.003	0.024 ± 0.001
	Stem	0.033 ± 0.007	0.028 ± 0.005	0.027 ± 0.001
	Leaf	0.026 ± 0.004	0.028 ± 0.000	0.027 ± 0.003
	Fruit	0.029 ± 0.002	0.027 ± 0.005	0.026 ± 0.003
**As**	Root	0.028 ± 0.008	0.041 ± 0.013	0.035 ± 0.016
	Stem	0.003 ± 0.001	0.005 ± 0.002	0.005 ± 0.002
	Leaf	0.011 ± 0.003	0.018 ± 0.008	0.013 ± 0.010
	Fruit	0.001 ± 0.000	0.001 ± 0.001	0.003 ± 0.004

Note: LD, MD, and HD denote low density (2 plants pot^{-1}), medium density (6 plants pot^{-1}), and high density (10 plants pot^{-1}), respectively. Data are presented as mean ± standard deviation (SD). Outliers arising from experimental or instrumental anomalies have been appropriately excluded to ensure statistical accuracy. No statistically significant differences were observed among the LD, MD, and HD treatments across all evaluated heavy metals ($p > 0.05$).

Table S4. Translocation factors (TF) of heavy metals in *D. stramonium* separated by individual planting density treatments.

Treatment	Pb	Cd	Cr	As
**LD**	1.599 ± 0.272	0.247 ± 0.079	0.146 ± 0.158	1.827 ± 0.482
**MD**	1.829 ± 0.908	0.185 ± 0.041	0.143 ± 0.165	1.865 ± 0.926
**HD**	1.897 ± 1.170	0.322 ± 0.141	0.277 ± 0.037	2.669 ± 1.789

Note: LD, MD, and HD denote low density (2 plants pot^{-1}), medium density (6 plants pot^{-1}), and high density (10 plants pot^{-1}), respectively. Data are presented as mean ± standard deviation (SD). No statistically significant differences were observed among the LD, MD, and HD treatments across all evaluated heavy metals (One-way ANOVA, $p > 0.05$), indicating that the translocation capacity is a highly conserved physiological trait unaffected by planting density.

Table S5. Concentrations of heavy metals in different organs of *D. stramonium* separated by individual planting density treatments.

Heavy Metal	Organ	LD (Mean ± SD)	MD (Mean ± SD)	HD (Mean ± SD)
Pb	Root	1.476 ± 0.233	2.265 ± 0.952	1.858 ± 0.976
	Stem	0.407 ± 0.252	0.376 ± 0.168	0.352 ± 0.079
	Leaf	0.388 ± 0.128	0.811 ± 0.284	0.589 ± 0.267
	Fruit	0.168 ± 0.117	0.110 ± 0.066	0.150 ± 0.081
Cd	Root	0.409 ± 0.153	0.394 ± 0.079	0.408 ± 0.105
	Stem	0.634 ± 0.394	0.737 ± 0.239	0.611 ± 0.318
	Leaf	1.085 ± 0.613	1.340 ± 0.572	1.011 ± 0.663
	Fruit	0.204 ± 0.053	0.162 ± 0.059	0.237 ± 0.054
Cr	Root	1.701 ± 0.235	1.510 ± 0.204	1.504 ± 0.164
	Stem	2.147 ± 0.393	1.807 ± 0.253	1.852 ± 0.162
	Leaf	1.812 ± 0.345	1.997 ± 0.195	1.782 ± 0.146
	Fruit	1.824 ± 0.231	1.903 ± 0.256	1.820 ± 0.240
As	Root	0.876 ± 0.277	1.335 ± 0.423	1.100 ± 0.471
	Stem	0.108 ± 0.031	0.167 ± 0.082	0.156 ± 0.072
	Leaf	0.350 ± 0.106	0.595 ± 0.276	0.411 ± 0.350
	Fruit	0.032 ± 0.008	0.041 ± 0.040	0.027 ± 0.008

Note: LD, MD, and HD denote low density (2 plants pot^{-1}), medium density (6 plants pot^{-1}), and high density (10 plants pot^{-1}), respectively. Data are presented as mean ± standard deviation (SD). Units are mg/kg.
